# NewAbstractConcepts: A Database of 42 Normed Abstract Concepts and Exemplars

**DOI:** 10.5334/joc.384

**Published:** 2024-07-12

**Authors:** Dounia Lakhzoum, Marie Izaute, Ludovic Ferrand, René Zeelenberg, Diane Pecher

**Affiliations:** 1 Université Clermont Auvergne, CNRS, LAPSCO, F-63001 Clermont-Ferrand, France; 2 Department of Psychology, Education, and Child Studies, Erasmus University Rotterdam, The Netherlands

**Keywords:** abstract concept, concept learning, generalization mechanisms, abstraction, similarity, contextual diversity

## Abstract

Recently, researchers have expressed challenges in conducting word-learning experiments in adult populations due to limited availability of normed stimulus materials. This constraint often prompts the use of low-frequency or low-prevalence words, introducing the potential influence of prior knowledge or direct translation to familiar words. In response, we developed novel abstract concepts devoid of word referents, providing better control over prior knowledge. These new concepts describe situations encountered in various settings for which there is no existing word in English. The resulting database comprises 42 normed New Abstract Concepts, offering unique materials structured through scenarios, each containing similar and dissimilar exemplars. These materials underwent meticulous norming for relatability and similarity levels across a series of studies. The success of our approach was demonstrated in a word-learning experiment examining the effects of similarity and diversity. The database serves as a valuable resource for selecting stimuli in experiments exploring the learning of abstract semantic concepts, particularly investigating the role of similarity versus diversity in concept learning.

The database is available on OSF (https://osf.io/svm2p/).

The capacity to derive meaningful representations from a few exemplars and apply them to novel situations is the hallmark of human cognition. The existing literature primarily employs concrete stimuli, such as geometrical shapes, objects, animals, and faces, limiting our understanding of how abstract semantic concepts are acquired and generalized specifically as regards the role of similarity vs. diversity in concept learning.

## Types of stimuli in similarity-based concept learning

Various theoretical perspectives have contributed insights into the role of similarity in learning new concepts. Prototype and exemplar accounts have primarily relied on concrete stimuli such as shapes, objects, and animals, to investigate fundamental principles of similarity-based concept learning (Medin & Schaffer, 1978; Rosch et al., 1976; Medin et al., 1993). For instance, studies using animal stimuli have demonstrated concept organization through prototypicality, where a robin is considered more representative of the bird category than a penguin (Rosch et al., 1976). Other studies have used geometrical shapes to demonstrate exemplar-based similarity wherein previous encounters with specific shape exemplars can influence subsequent category decisions (Medin & Schaffer, 1978). Concepts often integrate situational elements rather than focusing on individual concrete objects. This requires the use of complex materials such as situations or scenarios to investigate novel concept learning. Gentner and Markman (2006) showed that participants used situations that reflected meaningful relational similarity rather than superficial similarities to make inferences. More recently, Cho et al. (2024) investigated neural processing of visual qualities, in terms of prototypicality and simplicity, when forming design preferences and showed that prototypical designs are processed more easily and judged as more aesthetically pleasing compared to non-prototypical designs. Similarly, Haro et al. (2023) explored emotional prototypicality, which indicates the extent to which a word refers to an emotion and found that emotional prototypicality positively influenced the recognition of emotion-label words. These recent developments emphasize the role of prototypicality in engaging higher-level semantic processing.

While language acquisition has historically posed challenges for general learning theories, studies using language stimuli emphasize the significance of analogical comparison (Gentner, 1989). Gentner, Loewenstein, and Hung (2007) tested structural alignment in children’s ability to name parts of objects, finding that a set of similar exemplars facilitated the structure-mapping process (Gentner & Gunn, 2001; Gentner & Markman, 2006) therefore strengthening the ability to generalize common relational features to new objects and situations (Gentner, Anggoro, & Klibanoff, 2011; Gentner, Loewenstein, & Hung, 2007; Kotovsky & Gentner, 1996; Namy & Gentner, 2002). Most studies on similarity and concept learning primarily involve infants and children because their limited background knowledge facilitates the development of stimulus materials and interpretation of results. However, there is a lack of similar studies with adult learners. In one such study, Bassok and Medin (1997) presented adult participants with noun-verb-noun statements to investigate structural alignment.

Similarity among stored exemplars does not always facilitate generalization. Rather, a substantial body of research suggests that an excessive degree of similarity impedes the capacity to generalize to new instances of a concept (Braithwaite & Goldstone, 2015; Gentner & Bowdle, 2008; Gentner, Loewenstein & Thompson, 2003). Notably, this effect seems to be qualified by the number of similar exemplars, as illustrated by Poch et al. (2019), who found that the accumulation of similar interfering experiences hinders the ability to retrieve information, leading to increased mnemonic interference.

## Types of stimuli in diversity-based concept learning

Posner and Keele’s (1968) influential work based on dot pattern classification suggested that the accuracy of classifying new instances improved with the variability of memorized patterns. The hypothesis of generalization from stored exemplars to novel instances posits the presentation of common core features between exemplars, allowing idiosyncratic surface features to vary (Belenky & Schalk, 2014; Day & Goldstone, 2012; Gentner, 1983; Gick & Holyoak, 1983, Goldstone, 1994). This process ensures that diverse superficial features are considered anecdotal, allowing learners to focus on the essential meaning of a concept and disregard extraneous details (Braithwaite & Goldstone, 2015). For instance, in presenting face-name pairs in adults, Smith and Handy (2014) found that acquisition trials presented in varied background contexts led to memory retrieval independently of context. Braithwaite and Goldstone (2015) demonstrated that altering superficial features of mathematical problem exemplars enhanced transfer abilities for expert participants, whereas novices benefited from similar superficial features.

For reasons mentioned earlier, studies exploring the influence of contextual diversity on word-learning have primarily involved infants and children. Studies have shown that exposing children to multiple exemplars of novel words across diverse contexts increases their ability to generalize meaning to novel test exemplars (Goldenberg & Sandhofer, 2013; Sandhofer & Schonberg, 2020; Twomey & Westermann, 2018; Vlach & Sandhofer, 2011). Rosa, Tapia, and Perea (2017) asked 8–9-year-olds to read unfamiliar words embedded in either same-themed or different-themed texts and showed that words encountered in different-themed texts were processed more effectively (but see Joseph & Nation, 2018).

Implementing a word learning paradigm with adult learners poses a challenge, primarily due to the potential influence of prior knowledge. Some studies have addressed this concern by employing low-frequency words. Johns et al. (2016) explored how novel words are represented in adult learners by exposing them to low-frequency target words within similar or different topical contexts. Similarly, Pagan and Nation (2019) investigated the impact of contextual diversity on adult participants learning low-prevalence words (Brysbaert et al., 2018). Participants were exposed to either four varied or identical sentences for each word, and in the test phase, they read the words in novel sentences. The presentation of identical sentences in the similarity condition, and the use of low-prevalence words introduce limitations, as participants may guess the familiar synonym. To reduce the influence of prior knowledge, Norman et al. (2023) used eight pseudowords instead of unfamiliar past-tense verbs. Similarly, Hulme et al. (2023) used sixteen novel pseudowords with corresponding meanings, embedded in paragraphs with low or high contextual diversity. However, the use of pseudowords only partially mitigated the issue, as the base words were concrete nouns or verbs, potentially leading to associations with prior meanings. Furthermore, Gatti et al. (2023) found that pseudowords can activate semantic networks similarly to existing words. In a lexical decision task, they showed that despite being novel or unknown, these linguistic units can access stored lexical-semantic information, challenging the traditional view that pseudowords lack meaning.

Some studies have developed and employed more inventive approaches to study concept learning in adults. For instance, Espey et al. (2021) investigated the acquisition and representation of novel abstract concepts grounded in linguistic and emotional experiences. They developed a linguistic training paradigm aimed at inducing novel abstract concepts based on linguistic experience. This involved presenting participants with the names of novel concepts along with written definitions and example situations in which the concepts could occur. The novel abstract concepts were either neutral or emotional. Over five linguistic training sessions, participants learned the names and definitions of these concepts and engaged in either mental imagery or lexical rephrasing of example situations where the novel concepts could occur. The study found that participants successfully acquired and recognized the novel concepts induced by the laboratory-controlled linguistic experience. Additionally, participants produced more features for emotional novel concepts compared to neutral ones, reflecting an emotional enrichment of the novel abstract semantic representations. This study further illustrates the extensive work required in the development of materials to investigate fundamental factors that play a role in word learning and, more generally, concept acquisition.

## Limitations of word-learning studies and contribution of the present database

Recently, researchers have expressed challenges in conducting word learning experiments with adults (Hoffman & Woollams, 2015; Jones et al., 2017; Norman et al., 2023; Rosa et al., 2017; Joseph & Nation, 2018). First, word learning is difficult to implement in adult learners due to the possible influence of prior knowledge. Additionally, the issue of word learning studies is marked by inconsistent definitions and operationalizations. Similarity and variability are not systematically operationalized across studies with some studies consider similarity as a single discourse topic (Johns et al., 2016; Joseph & Nation, 2018; Mak et al., 2021), while others use repetition of the same material (Bolger et al., 2008; Pagan & Nation, 2019). The criteria for high diversity also lack clarity. For instance, there is a discrepancy in the number of topics used in different experiments, raising questions about what constitutes high contextual diversity. Another limitation of word learning paradigms is the restricted number of exposures participants typically receive due to the restricted number of items that are generated in each experiment (see Norman et al., 2023 for a discussion).

In response to these challenges, the present database fills a crucial gap in concept learning research by introducing 42 New Abstract Concepts, meticulously normed and validated through a word learning experiment with native English adult learners. Unlike most adult concept learning studies employing pseudowords based on low-prevalence or low-familiarity words, our approach introduced novel abstract concepts devoid of word referents, offering control over background knowledge. These novel abstract concepts illustrate situations encountered in various settings but for which no known word exists (e.g., “*A hypothetical conversation that you compulsively play in your head”*), representing a departure from the lexicon’s existing vocabulary. These concepts were articulated through scenarios, each comprising similar and dissimilar exemplars, normed for relatability and levels of similarity across a series of studies.

## Summary of Approach

*Concept Selection:* We aimed to restrict the database to concepts that individuals had either experienced or could imagine ensuring relatability of the materials. Relying on experimenter intuition, concepts were chosen from Skurnick’s (2016) book “That should be a word.” Each concept depicted abstract situations or feelings that people could relate to despite lacking a corresponding word in the vocabulary (e.g., A hypothetical conversation that you compulsively play in your head; The realization that each passer-by has a life as vivid and complex as your own; Using incorrect words that still get the point across).

*Scenario/Exemplar Norming:* For each concept, three distinct scenarios were generated, defining various aspects such as location, activity, and actors. For each scenario, three similar exemplars were created. These exemplars were then systematically rearranged, intermingling scenarios to create dissimilar between-scenario combinations. This process ensured within-scenario similarity and between-scenario diversity. The norming studies presented similar and dissimilar exemplars from each concept in pairs to assess the similarity of scenarios within the same concept and to confirm that the dissimilar scenarios were indeed perceived as distinct.

*Main Experiment:* We aimed to test the validity of stimuli for use in a word learning study. Participants were presented with new words (pseudowords) without definitions. Instead, they were shown three exemplars of situations in which the new word could apply, allowing them to extract the meaning of the pseudoword from the context provided by the exemplars.

## Norming Studies – Generating the Database

### Method

#### Participants

In total, 263 participants (72 males; *M_age_* = 25.86, *SD* = 5.58) took part in the norming studies presented online using Qualtrics (2023). They all gave their informed consent before taking part in the study. They received course credit or £3.80 for their participation. The study procedure was in line with the local ethics committee.

#### Materials and Procedures

**Selection of New Abstract Concepts** – For each concept, 80 participants (6 males; *M_age_* = 20.55, *SD* = 4.99) engaged in a three-alternative forced-choice questionnaire, indicating if a) they had experienced, b) could imagine, or c) neither for the described situation. Subsequently, participants provided examples to demonstrate understanding. Each participant saw 20 concepts randomly presented in a self-paced session of approximately 30 minutes.

Following participant assessments, 80% of the trials revealed that individuals either experienced or could imagine the presented concept. Concepts resulting in a 25% or higher rate of non-relatability (26 out of 76) were excluded. This elimination process resulted in a final set of 50 relatable concepts.

**Scenario generation and exemplar creation –** For each of the 50 concepts, three unique scenarios were created, defined by factors like location, activity, and actors. Each scenario was then detailed by three distinct exemplars of statements sharing similarities in terms of location, activity, etc. This process yielded a total of nine exemplars to illustrate each concept (see Table 1 for an example).

**Table 1:** Examples of Scenarios and Exemplars for the concept described as “The realization that each passer-by has a life as vivid and complex as your own.”


SCENARIO	EXEMPLAR	DESCRIPTIVE STATEMENTS

1 (stranger on a train)	A	On the train, he looked at the woman in the opposite seat as she opened her laptop and wondered what type of work she might do.

1	B	The man on the train was looking at sheet music and humming to himself and she thought he might be a musician travelling all over the country to give concerts.

1	C	She kept thinking that the woman sitting across the aisle on the train might be a scientist because she was studying papers with complicated looking graphs.

2 (a colleague speaks a different language)	A	Listening to her colleague answer the phone in his mother tongue, she suddenly realized that he grew up in a tropical country.

2	B	She heard the office manager talk to his children in a foreign language and started daydreaming about how their upbringing might have differed from her own.

2	C	When he heard the team manager answer her phone in fluent Japanese, he imagined what it was like growing up in the Japanese culture.

3 (teacher in a private setting)	A	It was strange to see the math teacher in the dairy section, but he realized that the teacher must have a family too.

3	B	At the concert he became aware of the coach’s private life when he saw him dressed for a party rather than in his usual sports outfit.

3	C	When the history teacher proudly told them he ran the New York City marathon, they knew he must have spent a lot of his free time training.


Subsequently, these exemplars were rearranged to create dissimilar between-scenario combinations (e.g., 1 A/1B/1C for similar scenarios and 1 A/2C/3B for dissimilar scenarios where numbers represent scenarios and letters represent exemplars). Exemplars were subsequently reorganized by pairs – either similar or dissimilar and tested in a paired similarity judgments paradigm.^1^

The materials comprised 900 pairs across 16 blocks, each containing 6–7 concepts (54–63 pairs per block). Seventy-one native speakers (32 males; *M_age_* = 27.01, *SD* = 5.13) were randomly assigned to a block, ensuring exposure to only one condition per concept (within-scenario or between-scenarios pairs). The order of pairs within each block was randomized, and participants rated the extent of similarity on a 0 to 100 scale for each pair presented sequentially on the screen using Qualtrics. The self-paced study took approximately 20 minutes to complete.

The aim was to ensure the constructed pairs accurately reflected within-scenario and between-scenario conditions. Concepts with higher similarity ratings for within-scenario pairs than between-scenario pairs were retained. Despite an overall tendency for within-scenario pairs to receive higher ratings (*M* = 70.08; *SD* = 10.85) than between-scenario pairs (*M* = 44.79; *SD* = 14.55; Mean Difference = 25.29), a qualitative analysis identified 25 concepts with a reversed pattern. These were reworked and presented to a new cohort of 112 participants (34 males; *M_ag_*_e_ = 30.01, *SD* = 6.63). Eight concepts with a reversed pattern were removed, and comparisons were based on the remaining 42 concepts. The constructed within-scenario pairs elicited higher similarity ratings (M_similar_ = 72.80, *SD* = 10.10) compared to between-scenario pairs (M_Dissimilar_ = 40.08, *SD* = 12.13; Mean Difference = 32.72), indicating improved statistics and a robust combination of materials. The materials were then tested in an empirical study of similarity and contextual diversity in concept learning.

## Main Experiment – Testing the Materials in a Word-learning Experiment

The materials from the database were used to explore the impact of contextual similarity in a word learning experiment in adult learners. This experiment aimed to highlight the advantages of utilizing novel abstract concepts to address concerns raised in recent studies. Firstly, the use of novel abstract concepts helps mitigate the confounding effect of prior knowledge. Moreover, the strength of the materials lies in their number, addressing the typical limitation of a restricted number of items in word learning experiments. Lastly, the within-scenario coherence and between-scenario diversity enhance the operationalization of variables for a more robust experimental design.

### Method

#### Participants

Sixty-three British native speakers were recruited online using Prolific to take part in this experiment (16 males; *M*_age_ = 30.29; *SD* = 8.29) presented using Qualtrics (2023). They all gave their informed consent before taking part in the study. They received £3.80 for their participation. The study procedure was in line with the local ethics review board.

#### Materials and Design

The concepts were organized into six lists. On each list, all concepts were presented with three exemplars, either similar or dissimilar (half of the concepts in each condition), such that across lists, all concepts were presented three times in the similar and three times in the dissimilar condition and each exemplar was presented once in the similar and once in the dissimilar condition. Participants were randomly assigned to one list, ensuring they saw only one set of similar or dissimilar examples for each concept. All participants experienced both similar and dissimilar conditions during the study.^2^

For each concept, a pseudo-word based on the English language was generated using Wuggy (Keuleers & Brysbaert, 2010) to serve as a referent label (e.g., *Remation* or *Unglith*). For each concept, a test exemplar corresponding to a novel scenario composed of a single exemplar was constructed (e.g., “*When he entered the living room, he saw a picture on a mantelpiece and was astonished that his neighbor had apparently met Bill Gates*”). Nonsensical filler sentences were also created to serve as attention check during the reading phase. These fillers followed the same rules as the actual exemplars but contained unrelated words, creating a sense of semantic ambiguity (e.g., “*Getting upstairs, she could not remember whether she had poisoned the front door*”). The experiment included 18 fillers, each presented with a pseudo-word generated using Wuggy as a referent.

#### Procedure

During the reading phase, participants were presented with a pseudo-word (e.g., REMATION) and three exemplars, either similar or dissimilar, presented one by one on the screen. Participants were not given definitions for the concepts but were instructed to focus on the pseudo-word and corresponding exemplars illustrating their meaning. Nonsensical sentences were randomly inserted to ensure participants’ attention. After each exemplar, participants selected the “next” button for the next trial or the “nonsensical” button if they identified a nonsensical sentence. Items were presented in six study-test blocks, each containing seven concepts with three sentences each. The presentation of exemplars and pseudo-words was randomized within each block. After each study block, participants entered a testing phase.

During testing, participants evaluated a novel test exemplar constructed as part of a fourth scenario for each concept. Test exemplars were presented with their corresponding pseudo-words, and participants rated on a scale from 0 to 100 whether the novel exemplar was a good representation of the pseudo-word.^3^ The self-paced study took approximately 30 minutes to complete.

## Results and Discussion

**Figure 1:**
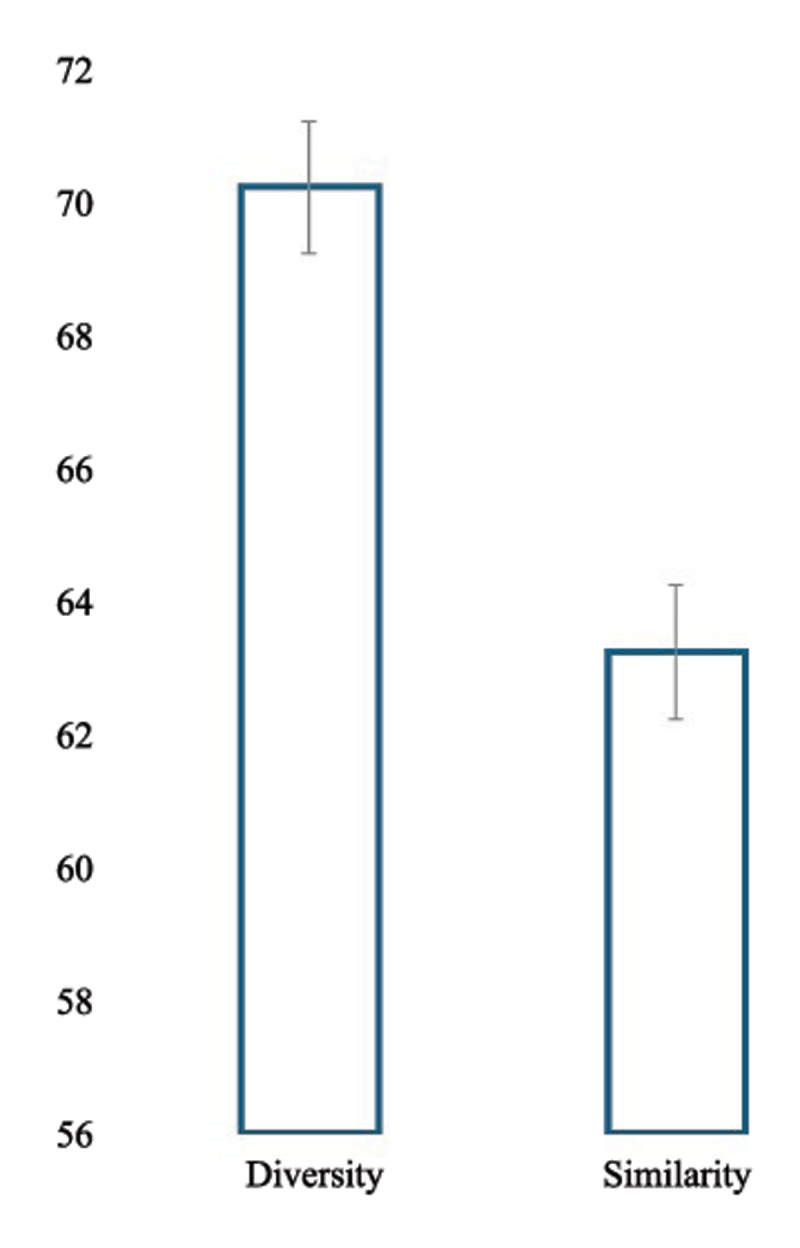
Mean Similarity judgments according to reading phase exposure (Similarity vs. Diversity).

To analyze the effect of Similarity vs. Contextual Diversity, we conducted a linear mixed-effects model (LME; Bates et al., 2015) accounting for items and participants as random effects, with the following structure: Rating∼Similarity_Condition+(1∣Ppt)+(1∣Item). Results showed that the test exemplars were judged as better examples of the concepts when participants were exposed to dissimilar exemplars during the reading phase (*M_Dissimilar_* = 70.27; *SD* = 25.40) compared to when they were exposed to similar exemplars (*M_Similar_* = 63.07; *SD* = 29.02; see Figure 1). Specifically, the average rating for the Similar condition was 7.23 points lower than the Dissimilar condition (*z* = –7.52, *p* < 0.001; see Table 2).

**Table 2:** Results of the LME analyses on Similarity Ratings.


EFFECT	*β*-ESTIMATE	*SE*	*Z*	*95% CI*	*p*

Intercept	70.28	1.73	*40.59*	66.89–73.68	<0.001

Condition Similarity [Similar]	–7.23	0.96	*–7.52*	–9.11––5.34	<0.001


*Note*. LME = linear mixed effects, *SE* = standard error.

These results indicated that exposure to contextually diverse exemplars led to improved performance in the testing phase. This outcome suggests that contextual diversity during reading may contribute to the development of more context-independent word representations (e.g., Bolger et al., 2008; Pagan & Nation, 2019).

These results affirm the database’s utility in examining the impact of similarity versus diversity on word learning in adults, addressing challenges raised by recent studies. We avoided the confounding effect of prior knowledge, and the ample number of items eliminates the need for repetition.

## General Discussion

The present studies addressed previous issues by introducing a database of normed novel abstract concepts. This innovative approach reduces the influence of background knowledge and provides stimuli for studying mechanisms of concept learning, abstraction, and generalization in abstract concepts in adults, filling a crucial gap in the word-learning literature. Notably, the database offers a substantial number of stimuli, surpassing typical word learning tasks, and ensures clear definitions of similarity and dissimilarity with normed similarity ratings.

The materials were successfully tested in a word-learning experiment with adult learners. We found improved concept learning when studied exemplars were dissimilar to each other. These findings align with previous research emphasizing the role of diversity in generalization. Broadly, when irrelevant features are dissimilar between exemplars learners find it easier to identify relevant common core features (Belenky & Schalk, 2014; Day & Goldstone, 2012; Gentner, 1983; Gick & Holyoak, 1983).

In our study, we normed various aspects of the stimuli to ensure their effectiveness and reliability. We selected relatable concepts and created scenarios and exemplars that maintained within-scenario similarity and between-scenario diversity. This comprehensive norming process enables users of the database to confidently use the materials. However, one limitation of this approach is that we did not specifically ask participants whether they felt the exemplars accurately illustrated the target concepts. While the norming ensured that the scenarios were relatable and the exemplars were coherent and diverse, it did not directly assess the exemplars’ success in evoking the intended concepts. Despite this limitation, the results from the word learning experiment we conducted showed that participants were indeed able to extract the meaning of the novel concepts based on the exemplars we presented and confirmed the effect of contextual diversity on participants’ ability to generalize meaning.

Further investigations are required to fully understand the mechanisms underlying the acquisition of novel abstract concepts. By using our database and modifying the paradigm employed in the main experiment, researchers can explore numerous research questions and mechanisms:

For instance, they can further investigate how variations in contextual diversity during learning impact the ability to generalize abstract concepts. This could involve presenting words in multiple contexts (or scenarios) and measuring individuals’ ability to recall and apply the concepts in new, unsampled contexts. Additionally, researchers can compare the effects of distributed learning (spacing intervals between study sessions) versus massed learning (cramming) on the retention and recall of abstract concepts. Studies can also explore how repeated exposure to abstract concepts enhances memory retention by measuring recall performance after varied numbers of repetitions to identify the optimal number for effective learning. Furthermore, researchers can examine how prior knowledge influences the learning process by comparing novel concepts devoid of existing word referents to known abstract concepts. The effect of different types of instructions, such as direct instruction versus discovery-based learning, can be assessed to determine their effectiveness in learning novel abstract concepts. By manipulating the cognitive load in the task, researchers can investigate its impact on learning efficiency and retention. Finally, exploring how cultural and linguistic backgrounds influence the learning and generalization of novel abstract concepts can offer valuable insights into the universality of concept acquisition, including the learning of novel abstract concepts in second language acquisition.

The adaptable nature of the database also allows for potential expansions, considering additional dimensions or varying task types such as similarity judgment tasks (how individuals judge the similarity between learned and novel exemplars), production tasks (studying individuals’ ability to produce examples of the learned concepts), interpretation tasks (how individuals interpret new situations using the learned abstract concepts) and metacognitive judgments (how individuals evaluate their own learning and understanding of novel abstract concepts).

To conclude, this database aims to fill a gap in the literature, providing a valuable resource for studying the generalization of semantic abstract concept learning in adults, an area that has been relatively unexplored.

## Data Accessibility Statement

The database is available on OSF at: https://osf.io/svm2p/.

## Footnotes

A table of the similar-dissimilar combinations is available on OSF: https://osf.io/qwz4t.

Details of the materials and experiment blocks are available on OSF: https://osf.io/c8mwf.

We assessed the reliability of the scores on the 0–100 scale using the split-half method (Spearman-Brown prediction formula). Results showed a correlation of r = .88, indicating a high level of internal reliability.
